# Learning curve using the Sunnybrook Facial Grading System in assessing facial palsy: An observational study in 100 patients

**DOI:** 10.1111/coa.13574

**Published:** 2020-06-08

**Authors:** Martinus M. van Veen, Tessa E. Bruins, Madina Artan, Paul M.N. Werker, Pieter U. Dijkstra

**Affiliations:** ^1^ Department of Plastic Surgery University Medical Center Groningen University of Groningen Groningen The Netherlands; ^2^ Center for Rehabilitation University Medical Center Groningen University of Groningen Groningen The Netherlands; ^3^ Department of Oral and Maxillofacial Surgery University Medical Center Groningen University of Groningen Groningen The Netherlands

**Keypoints**

1. Little is known about facial function assessments of inexperienced observers in facial palsy.
2. In this observational study, learning curve was examined of two inexperienced observers assessing facial function of 100 patients using the Sunnybrook Facial Grading System.
3. Interobserver agreement gradually improved over time, stabilising after approximately 70 assessments.
4. Best agreement on the voluntary movement subscore was observed, followed by synkinesis and resting symmetry subscores.
5. Inexperienced observers can perform facial function assessments in facial palsy, but should be adequately trained first.

## 1 INTRODUCTION

Assessment of facial function in facial palsy patients is important to evaluate current status and treatment effect. The Sunnybrook Facial Grading System (SB) is one of the clinician grading's of facial function.^1^ Inter‐ and intraobserver reliability ranges from 0.838 to 0.980 and 0.831 to 0.997, respectively.^2^ However, most reliability studies included experienced observers. In research projects, facial palsy assessment is often done by medical students. Additionally, general practitioners and starting residents or physical therapists may not have extensive experience in facial palsy assessment using the SB. Aim of this study was to analyse a learning curve for facial function assessment in facial palsy using the SB in a 7‐week prospective observational study with two inexperienced final‐year medical students.

## 2 MATERIALS AND METHODS

### 2.1 Ethical considerations

This study was performed at the University Medical Center Groningen, the Netherlands. No formal ethical review by the Institutional Review Board was required. All patients provided written consent prior to this study.

### 2.2 Procedure

Two medical students without previous extensive knowledge of facial palsy (TB and MA) participated in this 7‐week training period in March and April 2019 using the SB. Both students were final‐year medical students, approximately 6 months prior to obtaining their MD‐degree, having done two years of clinical rotations. Prior to the start, the students were informed of the criteria for grading the SB,^3^ watched the SB and eFACE tutorial videos (https://sunnybrook.ca/content/?page=facial-grading-system and http://links.lww.com/PRS/B355, respectively) and performed two SB assessments together with a researcher with experience in facial palsy grading (MMvV). Thereafter, the students independently watched 10 videos of facial palsy patients performing standard facial movements and performed a SB assessment. At the end of the week, a meeting was held in which the students and experienced researcher watched the videos and discussed disagreements. In an open dialogue, reasons for choosing a certain grading were shared and discussed, creating a platform for reflection and learning. In week two to seven, 15 sets of videos were assessed, reviewed after each week in a joint meeting, resulting in a total of 2 × 100 assessments. The learning curve was investigated by examining changes in interobserver agreement at the (sub)score and item level from week to week.

### 2.3 Sunnybrook Facial Grading System

The SB is a clinical grading system of facial function in facial palsy.^1^ It consists of 13 items—3 resting, 5 voluntary movement and 5 synkinesis items—answered on categorical answering scales. A SB composite score (range 0‐100) and resting symmetry (range 0‐20), voluntary movement (range 20‐100) and synkinesis subscores (0‐15) can be calculated.

### 2.4 Statistical analysis

Descriptive statistics were presented as numbers and frequencies, mean and standard deviation (SD) and median and interquartile range (IQR) when appropriate. For describing the sample, the mean SB scores of both observers were used. Interobserver agreement was analysed by calculating intraclass correlation coefficients (ICC, two‐way random effects model, single measures, absolute agreement) for the SB composite score and the three subscores. Item level interobserver agreement was assessed by calculating Cohen's κ statistic and percentage absolute agreement. For the first SB item (resting eye), an unweighted κ was calculated since categories are non‐ordered. For the second to thirteenth item, a quadratic weighted κ was calculated, since these items are ordered.

Additionally, we assessed final interobserver agreement between the two assessors of the last 50 SB assessments (videos 51‐100), since it is generally advised to perform reliability studies with at least 50 participants.^4^ A value of 0.7 was taken as an acceptable level of agreement for both the ICC and Cohen's κ, preferably for the lower border of the 95% CI.^4^

## 3 RESULTS

Videos of 100 individual patients with varying degrees of facial palsy were included. Fifty‐five patients were female, and the median (IQR) age was 45 (26; 56) years. Median (IQR) SB scores were 15.0 (10.0; 17.5‐range 5; 20) for resting symmetry, 42.0 (32.0; 50.0‐range 20; 72) for voluntary movement, 1.0 (0.0; 2.5‐range 0; 10) for synkinesis and 26.3 (16.1; 34.9‐range 0; 62) for the SB composite score.

**Figure 1:**
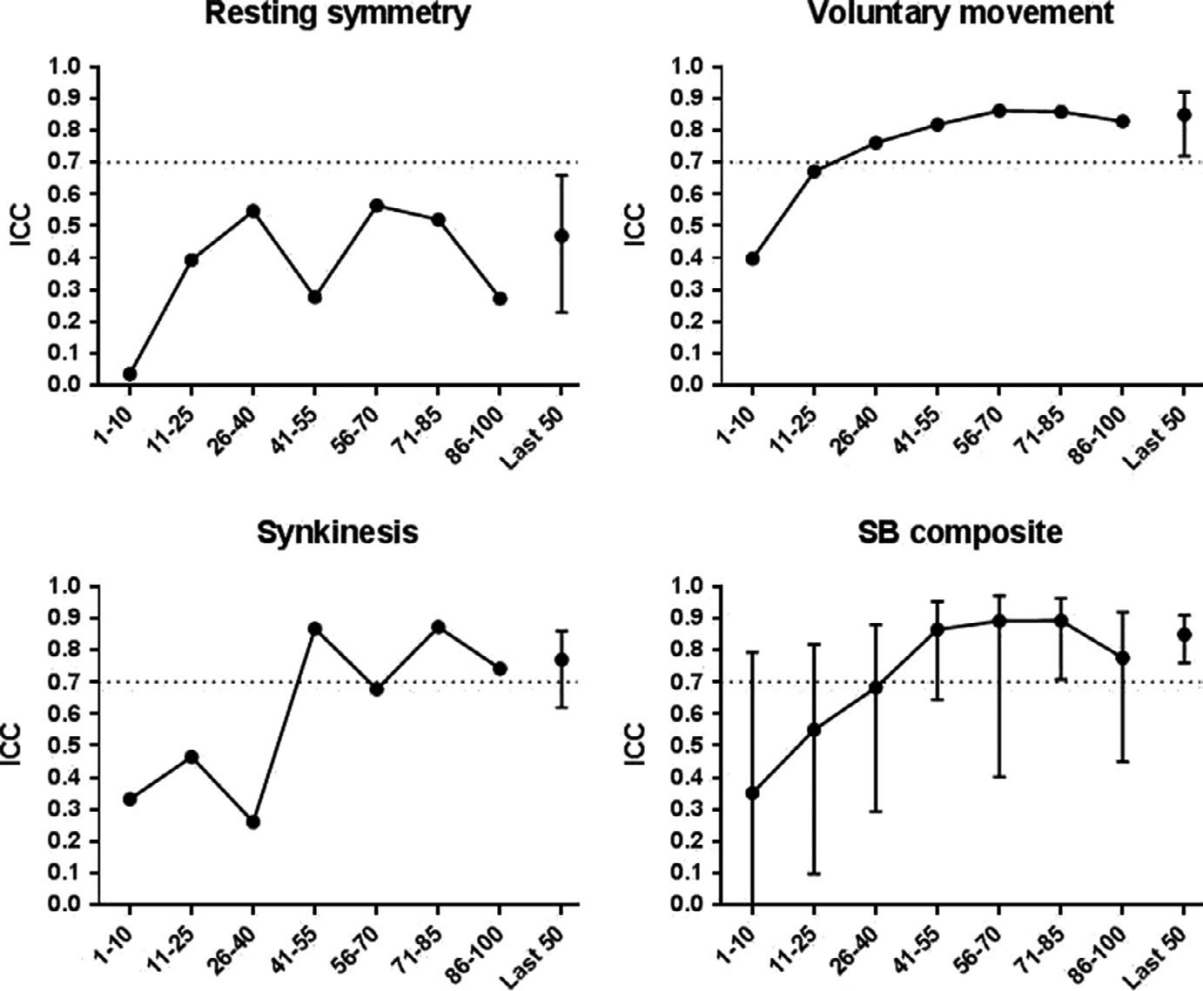
Graphical representation of interobserver agreement on Sunnybrook Facial Grading System (SB) (sub)scores over time. Circles with interconnecting line represent the intraclass correlation coefficient (ICC) for interobserver agreement for the videos in each of the 7 wk. 95% confidence interval is only presented for SB composite score, since is it the most important scale. Circle with error bars (*right*) represents the interobserver agreement on the last 50 videos (point estimate ICC and 95% confidence interval, respectively) on the last 50 videos. A horizontal line was placed at ICC = 0.7, the pre‐set acceptable level of agreement

Interobserver agreement for the SB (sub)scores gradually improved over time Figure 1). The steepest learning curve seemed to be for the SB composite score and voluntary movement subscore, followed by the synkinesis subscore. Similarly, interobserver agreement (ICC [95% CI]) on the last 50 patients was better for the SB composite score (0.85 [0.76; 0.91]) and voluntary movement subscore (0.85 [0.72; 0.92]), compared to the synkinesis subscore (0.77 [0.62; 0.86]) and resting symmetry subscore (0.47 [0.23; 0.66]).

Interobserver agreement for individual SB items improved gradually, but improvement differed per item (Table 1). Interobserver agreement on the last 50 patients was best for voluntary movement items (κ from 0.65 to 0.78), compared to resting items (κ from 0.58 to 0.80) and synkinesis items (κ from 0.38 to 0.73). In total, seven of 13 SB items scored Cohen's κ larger than 0.7 (Table 1).

**Table 1:** Cohen's κ values (% agreement) with quadratic weighting for individual Sunnybrook Facial Grading System items

Video	1‐10	11‐25	26‐40	41‐55	56‐70	71‐85	86‐100	Last 50 (51‐100)
Resting symmetry								
Eye^a^	0.51 (70%)	0.50 (60%)	0.45 (67%)	0.30 (47%)	0.41 (67%)	0.30 (47%)	0.56 (67%)	0.43 (58%)
Cheek	0.62 (50%)	0.56 (47%)	0.56 (47%)	0.65 (60%)	0.83 (67%)	0.74 (53%)	0.76 (73%)	0.80 (62%)
Mouth	0.22 (60%)	0.76 (80%)	0.71 (87%)	0.43 (67%)	0.52 (73%)	0.72 (67%)	0.48 (67%)	0.58 (72%)
Voluntary movement								
Brow lift	1.00 (100%)	0.61 (80%)	0.94 (87%)	0.94 (93%)	0.63 (93%)	1.00 (100%)	−0.07 (87%)	0.75 (92%)
Gentle eye closure	0.80 (60%)	0.80 (40%)	0.87 (53%)	0.61 (20%)	0.82 (40%)	0.75 (47%)	0.86 (67%)	0.78 (48%)
Open mouth smile	0.32 (40%)	0.78 (87%)	0.73 (60%)	0.60 (47%)	0.77 (60%)	0.81 (73%)	0.92 (73%)	0.77 (62%)
Snarl	0.13 (33%)	0.13 (33%)	0.70 (60%)	0.09 (60%)	0.81 (67%)	0.74 (80%)	0.75 (73%)	0.77 (74%)
Lip pucker	−0.07 (20%)	0.67 (60%)	0.59 (40%)	0.36 (13%)	0.63 (33%)	0.76 (60%)	0.58 (27%)	0.65 (40%)
Synkinesis								
Brow lift	0.78 (80%)	0.39 (60%)	0.12 (33%)	0.78 (80%)	0.81 (93%)	0.57 (73%)	0.81 (80%)	0.73 (82%)
Gentle eye closure	0.51 (50%)	0.64 (73%)	0.82 (87%)	0.75 (80%)	0.44 (73%)	0.84 (93%)	0.79 (87%)	0.73 (84%)
Open mouth smile	0.04 (40%)	0.10 (53%)	0.37 (73%)	0.72 (80%)	0.31 (73%)	0.59 (87%)	0.50 (67%)	0.51 (78%)
Snarl	0.33 (73%)	0.33 (73%)	—^b^ (67%)	—^b^ (87%)	—^b^ (93%)	—^b^ (87%)	0.40 (73%)	0.38 (86%)
Lip pucker	0.14^b^ (40%)	0.79 (73%)	—^b^ (80%)	0.68 (80%)	0.63 (93%)	1.00 (100%)	0.59 (87%)	0.67 (92%)

^a^ Unweighted kappa since this item is not ordered.

^b^ Cohen's κ could not be calculated since one observer scored all patients as “0,” and hence, a 2 × 2 table could not be formed.

## 4 DISCUSSION

### 4.1 Synopsis of key findings

Initial agreement between two inexperienced medical students was poorer and below any acceptable threshold for agreement than that of experienced observers. During the 7‐week training and feedback programme, they were able to reach acceptable interobserver agreement on the SB composite score and voluntary movement subscore; agreement on other subscores and individual items was lower. The ICC values of the last 50 observations remained lower than those reported for more experienced observers in literature.^1^, ^2^, ^3^, ^5^, ^6^

Although examining individual items should be done with caution—since the items are initially not individually validated and investigated—it was our impression that some items performed better than others. For example, “Brow lift” seemed relatively easy, while “lip pucker” seemed to be more difficult.

Contrary to our findings, previous publications including inexperienced observers reported adequate inter‐ and intraobserver reliability (ICCs > 0.819).^3^, ^5^, ^7^ Reasons for this difference could be that we used only two observers, or that these other studies have included the full range of SB composite scores thereby automatically increasing reliability. However, in our study most of the facial palsy patients were referred to our plastic surgery department for smile reanimation surgery. Therefore, SB score ranged on the lower end of the spectrum (SB composite score range: 0 ‐ 62) and most patients presented with flaccid facial palsy.

### 4.2 Strengths and limitations

We described the results of only one protocol. Therefore, we cannot draw conclusions on the optimal number of videos inexperienced observers should watch each week or the number and timing of feedback sessions. Future studies could focus on these questions in order to determine an optimal training protocol for inexperienced observers of facial function in facial palsy.

A limitation of this study was that we assessed only two observers. This number was a practical choice, since both students were performing a research project at our department at the same time, but limits generalisability to the general population of possible observers. Additionally—since the SB is subjective—we chose not to use expert scores as gold standard. Instead, disagreements were discussed with the experienced researcher. Including a “reference” score might have changed the results. Thirdly, the assessments were performed from a standardised video, instead of in‐person. Although video assessments have been shown to be reliable in experienced observers,^6^, ^8^ this may have been an extra barrier for our students. Actually, they reported ease of the SB assessment depended considerably on the quality of the videos. Additionally, the time to complete an SB assessment got considerably shorter over time, although a formal analysis could not be done since we did not collect these data.

Two limitations are perhaps due to the setting of the study. Our results are only valid for patients with a SB composite score range 0 to 62. Secondly, the “snarl” movement has previously not been incorporated in our standard video for facial palsy. Hence, scoring the “snarl” and its associated synkinesis asked quite some insight of our students, although this was not replicated in the interobserver agreement.

Lastly, we assessed interobserver agreement using ICCs and Cohen's κ statistics. Although correct, these tests highly depend on the distribution of scores.^4^ Since the theoretical range of scores for resting symmetry is much smaller than the range of scores for voluntary movement for example, the ICC for resting symmetry is automatically lower. Therefore, especially for the individual items, the κ can sometimes be very low, while the proportion agreement is still relatively high. This makes interpretation of our results a bit difficult.

### 4.3 Clinical applicability

The results of our study can be used in future studies in which medical students participate, when training starting residents, or when communicating with colleagues less exposed to facial palsy. We eye‐balled that there was little improvement of interobserver agreement from week 5 onwards. Therefore, we advise that inexperienced observers are supervised for at least 70 SB assessments of facial palsy before their assessments are considered to be adequate.

## 5 CONCLUSIONS

Our study shows that initial agreement on facial function assessment in facial palsy between inexperienced observers is low. During our 7‐week training period, agreement gradually increased to acceptable levels, especially for the SB composite score.

## Data Availability

Data are available on request from the authors.
